# Development of Novel Potentiometric Sensors for Determination of Lidocaine Hydrochloride in Pharmaceutical Preparations, Serum and Urine Samples

**Published:** 2017

**Authors:** Tamer Awad Ali, Gehad Genidy Mohamed, Ghada A. Yahya

**Affiliations:** a *Egyptian Petroleum Research Institute (EPRI), 11727, Cairo, Egypt.*; b *Chemistry Department, Faculty of Science, Cairo University, 12613, Giza, Egypt.*

**Keywords:** Modified screen-printed electrode, Modified carbon paste electrode, Lidocaine hydrochloride, Pharmaceutical preparation, Urine and serum

## Abstract

This article is focused on the determination of lidocaine hydrochloride as a local anaesthetic drug. A potentiometric method based on modified screen-printed and modified carbon paste ion-selective electrodes was described for the determination of lidocaine hydrochloride in different pharmaceutical preparations and biological fluids (urine and serum). It was based on potentiometric titration of lidocaine hydrochloride using modified screen-printed and carbon paste electrodes as end point indicator sensors. The influences of the paste composition, different conditioning parameters and foreign ions on the electrodes performance were investigated and response times of the electrodes were studied. The electrodes showed Nernstian response of 58.9 and 57.5 mV decade^-1^ in the concentration range of 1×10^-7^–1×10^-2^ and 6.2×10^-7^–1×10^-2 ^mol L^-1 ^for modified screen-printed and carbon paste electrodes, respectively. The electrodes were found to be usable within the pH range of 2.0–8.0 and 2.0-7.5, exhibited a fast response time (about 6 and 4) low detection limit (1×10^-7^ and 6.2×10^-7 ^mol L^-1^), long lifetime (6 and 4 months) and good stability for modified screen-printed (Electrode VII) and carbon paste electrodes (Electrode III), respectively. The electrodes were successfully applied for the determination of lidocaine hydrochloride in pure solutions, pharmaceutical preparation and biological fluids (urine and serum) samples. The results obtained applying these potentiometric electrodes were comparable with British pharmacopeia. The method validation parameters were optimized and the method can be applied for routine analysis of lidocaine hydrochloride drug.

## Introduction

Lidocaine hydrochloride (LCHC) or 2-diethylamine-N-(2,6-dimethylphenyl)-ethanamide hydrochloride (Scheme 1) was a local anesthetic drug that reversibly inhibits the nerve impulse transmission. It bound to the receptors in sodium channels and decreased their activity functioning as a cell membrane stabilizer. It had a good superﬁcial activity, penetrating in depth through the mucous membranes and reduced the sensation pain (1, 2). LCHC was characterized by a fast onset and an intermediate persistence of activity. Like other local anaesthetics, at relatively high plasma concentrations, lidocaine possessed relevant systemic adverse effects, mainly on the central nervous and cardiovascular systems (3, 4). When used for topical application, its absorption from the intact skin was poor. However, when applied to damaged skin, the systemic absorption can be more effective (5). Several approaches were developed to enhance the local anaesthetic permeation through the use of liposomes (6, 7).

Several analytical methods were described for the detection of local anaesthetics. These included colorimetry (8, 9), gas-liquid chromatography (10, 11) and high pressure liquid chromatography combined with UV detection (12) or electrochemical detection (13). Ion-selective electrodes (ISEs) reported for lidocaine (14) were all based on ion pairing. All electrodes produced a Nernstian response in aqueous solutions, however, few interference studies were performed and no protein interference effects were studied. In a previous publication (15), lipophilic cyclodextrins were reported as the first neutral ionophores for local anaesthetics (16) with excellent selectivities over charge dense cations and several endogenous cations.

The carbon paste electrodes (CPEs) were suggested as a very useful end point indicator electrodes in the potentiometric titration of drugs. In comparison with similar PVC and coated wire electrodes, CPEs had the advantages of very low Ohmic resistance, very short response time in addition to the ease of fabrication and regeneration as well as long functional lifetime (17-20). Handmade carbon paste (made of carbon powder and liquid binder) was soft noncompatible material and had to be packed into a special electrode holder.

The described sensor made by thick-film and planar technologies were employed for developing solid-state sensors having low cost, small size and high reproducibility (20-29). Screen-printing was especially recommended as simple and fast method for mass production of disposable electrochemical sensors. Thick-film technologies were predominantly used for fabrication of amperometric devices. pH sensors were one of the first types of potentiometric sensors investigated for possible implementation through thick-film technology (29). 

In this paper, new potentiometric sensors were introduced for selective determination of lidocaine hydrochloride (LCHC) drug in pure solutions, pharmaceutical preparation and biological fluids (urine and serum) samples. The method was based on the incorporation of β-cyclodextrine (β-CD) and sodium tetraphenylborate (NaTPB) as electroactive materials and o-nitrophenyloctyl ether (*o*-NPOE) or tricresylphosphate (TCP) as placticizers in lidocaine hydrochloride matrix. These sensors exhibited analytical characteristics with near-Nernstian sensitivity, low detection limit and therefore, were useful as end point indicator electrodes in potentiometric titrations of LCHC in pharmaceutical preparations and biological fluids.

## Experimental

*Reagents*

Analytical reagent grades were used in this study and some of them were used as such without any further purification. They included LCHC provided by Misr Company for Pharmaceutical Industry, Egypt. Glucose, sucrose, starch, maltose, lactose, fructose, glycine and chloride salts of chromium, barium, ammonium, cobalt, manganese, magnesium, calcium, zinc and copper were used as interfering ions. Bidistilled water was used throughout all experiments.

For making ISE , the following reagents were used: *o*-nitrophenyloctylether (*o-*NPOE) was supplied from Fluka, while di-n-octyl phthalate (DOP), dibutylphthalate (DBP) and dioctyl sebacate (DOS) were supplied from BDH. In addition, tricresylphosphate (TCP), polyvinylchloride (PVC relative high molecular weight) and graphite powder (synthetic 1–2 µM) were supplied from Aldrich and β-cyclodextrine (β-CD) was purchased from Merck.

*Pharmaceutical samples*

Lidocaine gel (sample 1; Adco/Swanco Company, AL Amireya, Cairo, Egypt), farco-Caine oint (sample 2; Pharco Company, Alexandria, Egypt) and lidosine oint (sample 3; Alex Company, Alexandria, Egypt). Each oint in all samples had 20 g LCHC.

*Apparatus*

Laboratory potential measurements were performed using Jenway 3505 pH-meter. Silver-silver chloride double-junction reference electrode (Metrohm 6.0726.100) in conjugation with different ion selective electrode was used. pH measurements were done using Thermo-Orion, model Orion 3 stars, USA. Prior to analysis, all glassware used were washed carefully with distilled water and dried in the oven before use.

*Procedures*

*Preparation of modified screen printed electrode (MSPE)*

MSPE was prepared by using a manual screen printer. An array of 12 electrodes was printed on a flexible X-ray film by forcing the prepared conductive ink to penetrate through the mesh of a screen stencil. A screen consisting of a heavy duty polyester fabric (I 003 M Sefar Pet 1000 with mesh count of 36) was pre-tensioned to ca 30 × 40 cm wooden frame. For the stainless steel template, steel sheet were pre-tensioned to a steel frame and contain grooves with the same electrode dimensions (21, 24-26, 28, 29). The homemade printing ink was prepared by thoroughly mixing the cyclohexanone-acetone mixture 1:1, as a solvent for the binding material with 450 mg of TCP, 1.25 mg polyvinyl chloride, 0.75 mg of the carbon powder and then 5-15 mg of 1:1 (w%) β-CD:TPB ionophore was added after stirring for 15 min, the ink was sonicated and applied for printing of the electrodes. The influence of the plasticizer choice on the electrode performances was studied as the electrode plasticized with TCP was compared with those plasticized with DBP, DOP, DOS and *o*-NPOE. The MSPEs were stored in a dry state at room temperature.

*Preparation of chemically modified carbon paste electrode (CMCPE)*

A 500 mg pure graphite powder and 5-15 mg of 1:1 (w%) β-CD:TPB ionophore were transferred to mortar and mixed well with plasticizer (0.2 mL of DOP, TCP, DBP, DOS or *o*-NPOE). The modified paste was filled in electrode body and kept in distillated water for 24 h before use (19, 20, 28, 30-33). A fresh surface was obtained by gently pushing the stainless-steel screw forward and polishing the new carbon-paste surface with filter paper to obtain a shiny new surface. 

*Calibration of the new MCPE* ***‎*** *and MSPE*

The new MCPE and MSPE were calibrated by immersion in conjunction with a reference electrode in a 25-mL beaker containing 2.0 mL acetate buffer solution of pH 5. Then 10 mL aliquot of LCHC solution of concentration ranging from 1×10^-7^ to 1×10^-2^ mol L^-1^ were added with continuous stirring and the potential was recorded after stabilization to ± 0.1 mV. A calibration graph was then constructed by plotting the recorded potentials as a function of -log [LCHC)]. The titration graphs will be used for subsequent determination of unknown LCHC concentration (22, 29, 30, 34).

*Tetraphenylborate solution (TPB-)*

 2-01×1mol L^−1^ NaTPB solution was prepared by dissolving 1811 mg into 500 mL distilled water, adjusted to pH 9 by adding sodium hydroxide and completed to the desired volume with water. The resulting solution was standardized potentiometrically against standard (2-01×1 mol L^-1^) thallium (I) acetate solution (35).

*Effect of pH*

Series of pH solutions ranging from 1-11 were prepared at constant LCHC ion concentration, i.e. (1×10^-3 ^and 1×10^-5 ^mol L^-1^). The pH variations were brought about by the addition of dilute acid (HCl) and alkali (NaOH) solution. The value of electrode potential at each pH was recorded and was plotted against pH. Acetate buffer (pH 5) was prepared to adjust the pH of the solution.

*Effect of temperature*

The effect of temperature on the performance of the modified SPE and CPE sensors was evaluated in a thermostat at different temperatures ranged from 20 to 60 °C*. *

*Interfering ions solutions*

A 10^−3^ mol L^−1^ standard solution each of glucose, sucrose, starch, maltose, lactose, fructose, glycine and chloride salts of chromium, barium, ammonium, cobalt, manganese, magnesium, calcium, zinc and copper were prepared by dissolving the proper weights into 100 mL bidistilled water.

*Determination of LCHC drug in pharmaceutical preparations*

Lidocaine hydrochloride solution was prepared by mixing the content of ointment (lidocaine gel, farco-caine oint and lidosine oint), with 25 mL bidistilled water, stirred for 15 minutes at room temperature, filtered and the solution was completed to the mark (100 mL) with bidistilled water. The LCHC content was determined using the proposed potentiometric method.

*Procedure for the determination of LCHC in human urine and serum samples*

Preparing blood samples: 0.25 mL of whole blood sample was vortex mixed with 0.5 mL acetonitrile. The samples were subsequently centrifuged and the supernatant transferred to clean labeled tubes, each containing acetate buffer of pH 5. 

Preparing urine samples: 0.2 mL urine was hydrolyzed and 35 μL of 0.1 mol L^-1^ sodium phosphate buffer pH 4 was added and then heating samples at 50 °C for 1 hour. The samples were then cooled to room temperature and 0.25 mL acetonitrile was added to precipitate the enzyme. The solutions were centrifuged, and the resulting supernatants transferred to clean labeled sample tubes, each containing acetate buffer of pH 5.

Urine or serum samples containing different lidocaine concentrations were prepared by adding known amounts of LCHC to 25 mL aliquots of blank urine samples of four volunteers, the LCHC -selective and reference electrodes were immersed and the LCHC concentration was determined by direct potentiometry using the standard addition technique.

## Results and Discussion

**Figure 1:**
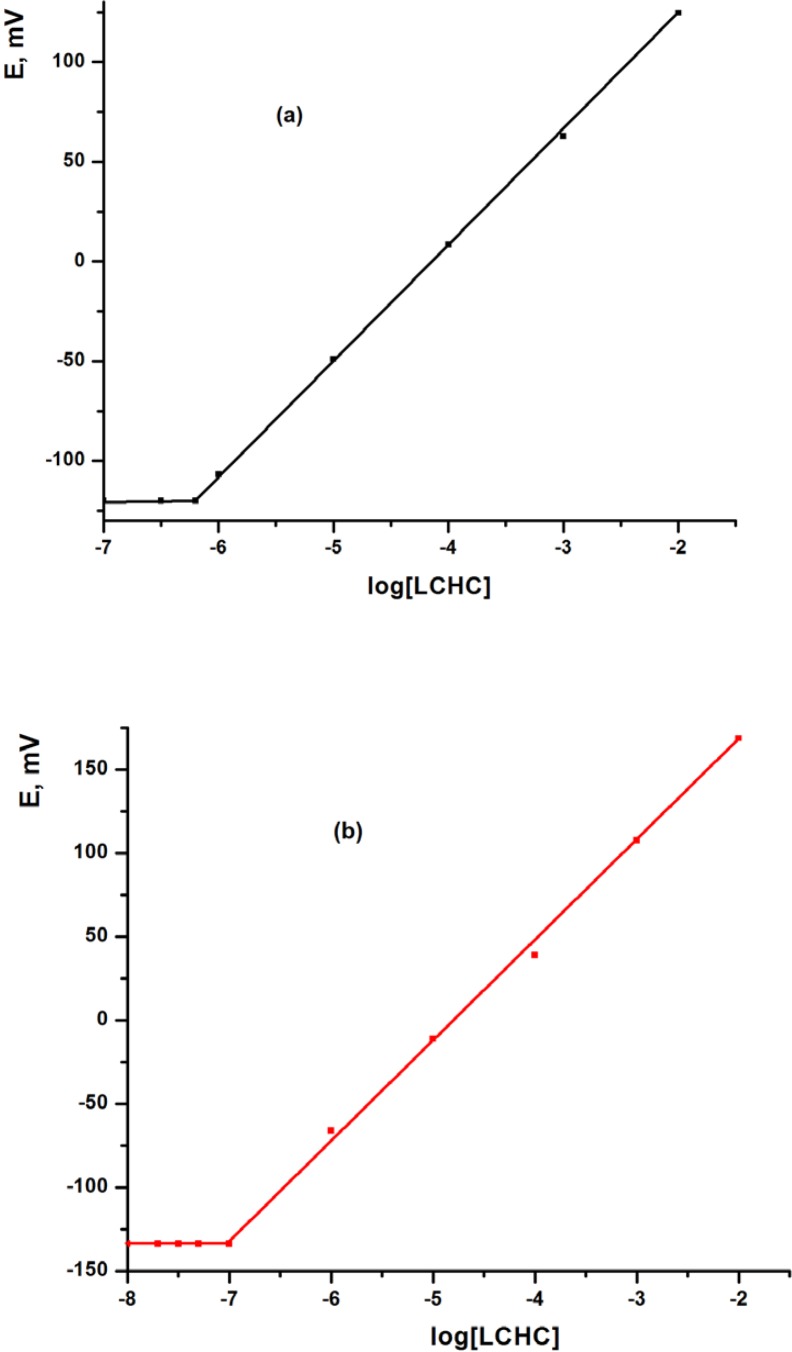
Calibration graphs using (a) MCPE and (b) MSPEs sensors using TCP plasticizer.

The MCPE and MSPE sensors were calibrated by transferring 10 mL aliquots of 1.0 × 10^-7^ - 1.0 × 10^-2^ mol L^-1^ aqueous solutions of LCHC to 25 mL beakers, followed by immersing the MCPE and MSPE sensors in conjunction with Ag/AgCl reference electrode in the solution. The potential readings were recorded after stabilization to ±1 mV and the e.m.f. was plotted as a function of p[LCHC] (Figure 1 and Table 1). The MCPE and MSPE showed a linear response over the concentration range from 6.2× 10^-7^ to 1.0 × 10^-2^ and from 1.0 × 10^-7^ to 1.0 × 10^-2^ mol L^-1 ^with Nernstian slope of 57.50 ± 0.89 and 58.90 ± 0.68 mV decade^-1^, respectively, using TCP plasticizer. The calibration graph was used for subsequent determination of unknown LCHC concentrations. 

*Effect of ionophore content*

**Figure 2:**
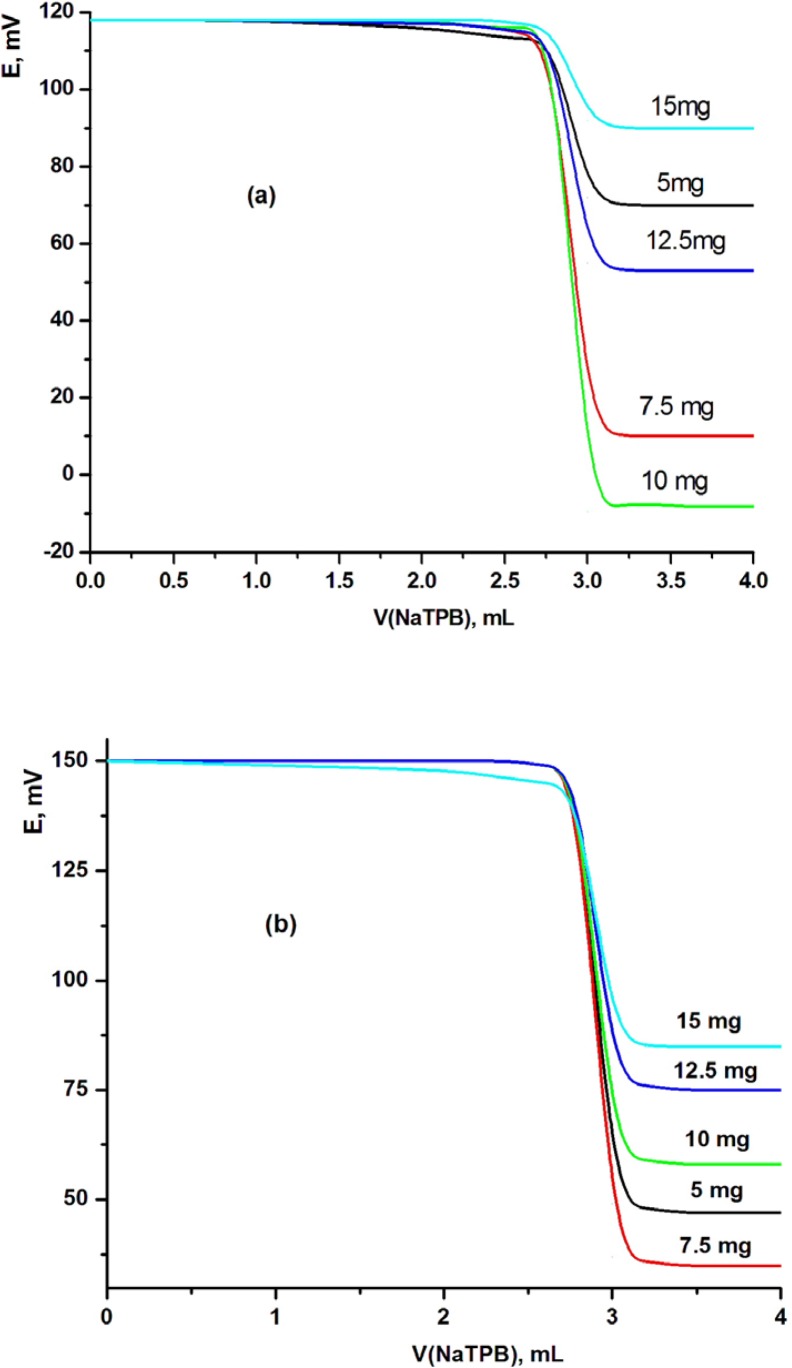
Effect of ionophore contents on (a) MCPE and (b) MSPE sensors using TCP plasticizer.

It is known that the sensitivity and linearity of a given electrode depend significantly on the amount of (β-cyclodextrine: tetraphenylborate) [β-CD:TPB] ionophore in the electrode composition. Thus, five MSPEs and MCPEs were prepared to determine the best electrode contents. The proportions of β-CD:TPB ionophore were varied as 5, 7.5, 10, 12.5 and 15 mg (w/w)%. The potentiometric titration was carried out for each electrode and the resulting potential breaks at the end point were found to be 41, 99, 125, 59 and 24, and 99, 111, 88, 71 and 57 mV mL^-1^ for modified MCPE and MSPE sensors, respectively. These electrodes gave sharp and reproducible inflection at the end point (125 and 111 mV mL^-1^ for modified CPE (electrode III) and SPE (electrode VII) sensors, respectively). These results indicated that the highest potential break at the end point was evaluated using 10 and 7.5 mg of [β-CD:TPB] ionophore for MCPE (electrode III) and MSPE (electrode VII) sensors, respectively. But when increasing the amount of ionophore over 10 and 7.5 mg, the total potential change decreased as shown in Figure 2. 

*Effect of the plasticizer type*

**Figure 3:**
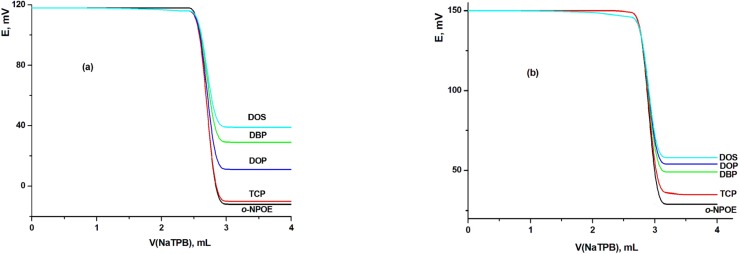
Effect of plasticizer type on the performance of (a) MCPE (electrode III) and (b) MSPE (electrode VII) sensors.

The influence of solvent mediator type and concentration on the characteristics of the LCHC-MCPE and LCHC-MSPE were investigated using five solvents with different polarities namely *o*-NPOE, TCP, DBP, DOP and DOS. The presence of plasticizers not only improved the workability of the sensor, but also contributed significantly to the improvement of the working concentration range, stability and life span of the electrode. The influence of the type of the plasticizer on the electrode performances was studied as the electrode plasticized with *o*-NPOE was compared with those of TCP, DOP, DBP or DOS. The obtained titration graphs with MCPE and MSPE using different plasticizers clarified that electrodes with *o*-NPOE and TCP as plasticizer showed the highest sensitivity and the best electrode was indicated with the highest total potential change and the highest potential break at the end point (Figure 3). Therefore, it can be concluded from this figure that two MCPEs (electrodes III and A using TCP and o-NPOE plasticizers, respectively) and two MSPEs (electrodes VII and B using TCP and o-NPOE plasticizers, respectively) gave the highest potential breaks at the end point and hence they selected for further studies. 

**Figure 4:**
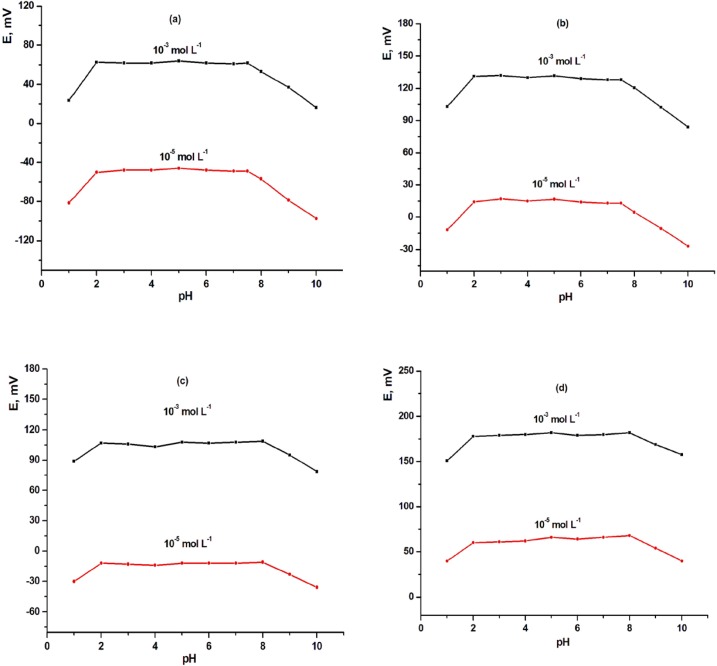
Effect of pH of the test solution on MCPEs [(a) electrode (III) and (b) electrode (A)] and MSPEs [(c) electrode (VII) and (d) electrode (B)].

**Figure 5:**
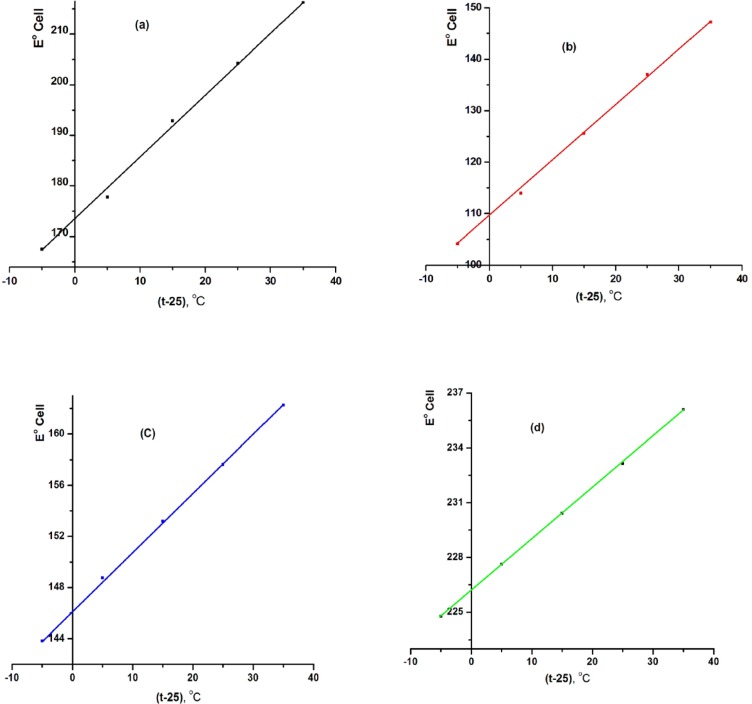
Effect of temperature on the performance of MCPEs [(a) electrode (III) and (b) electrode (A)] and MSPEs [(c) electrode (VII) and (d) electrode (B)].

**Figure 6:**
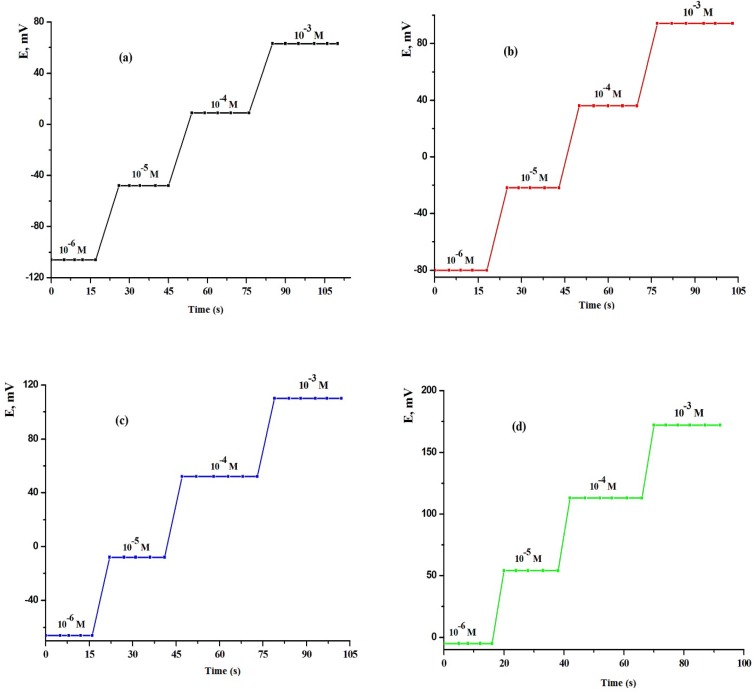
Dynamic response time of LCHC sensors of MCPEs [(a) electrode (III) and (b) electrode (A)] and MSPEs [(c) electrode (VII) and (d) electrode (B)].

**Figure 7:**
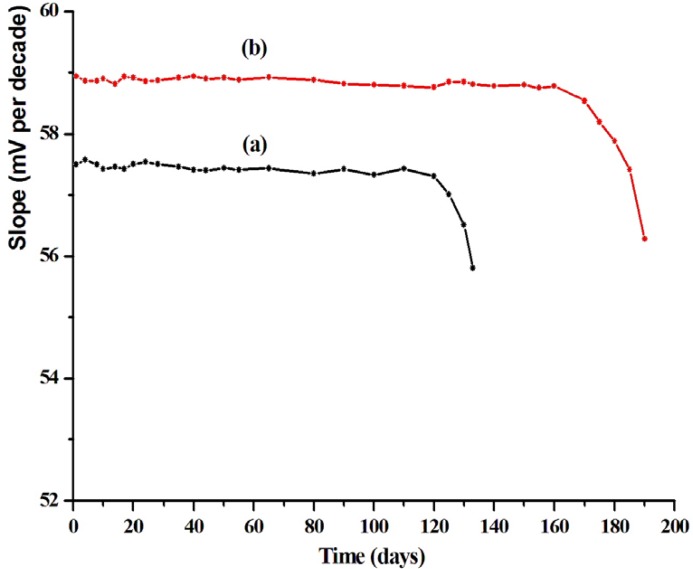
Life time of LCHC ion selective electrodes (a) MCPE [electrodes (III)] (b) MSPE [electrodes (VII)].

**Table 1:** Response characteristics of LCHC-MCPE (electrode III) and LCHC-MSPE (electrode VII) potentiometric sensors.

**Parameter**	** LCHC** **-MCPE ** **Electrode III**	**LCHC** **-MSPE** **Electrode ** **VII**
* Slope (mV decade^-1^)	57.50 ± 0.89	58.90 ± 0.68
Correlation coefficient, r	0.998	0.999
Lower detection limit (mol L^-1^)	6.2 × 10^-7^	1 × 10^-7^
limit of quantification (mol L^-1^)	14.45 × 10^-7^	3.33 × 10^-7^
Response time (s)	6	4
Working pH range	2 – 7.5	2 – 8.0
Usable range (mol L^-1^)	6.2×10^-7^ - 1×10^-2^	1×10^-7^ - 1.0×10^-2^
SD of slope (mV decade^− 1^)	0.207	0.110
* Intercept (mV)	238.42 ± 1.15	285.01 ± 1.03
Life time (months)	4	6
Accuracy (%)	99.74	99.86
Precision (%)	0.174	0.093

* The slope and intercept of the calibration curve

**Table 2:** Effect of soaking time on the performance of MCPE and MSPE potentiometric sensors.

	**MCPE (electrode III)**					**MSPE (electrode VII)**				
**Time of soaking**	**End point** **(mL)**	**Recovery (%)**	**Total potential change, mV**	**Potential break** **at the end point, mV**	**ΔE/ΔV** **(mV/mL)**	**End point** **(mL)**	**Recovery (%)**	**Total potential change, mV**	**Potential break** **at the end point, mV**	**ΔE/ΔV** **(mV/mL)**	
Without	2.99	99.67	127	125	312.5	2.97	99.00	113	108	275	
5 min	2.98	99.33	119	114	287.5	2.98	99.33	130	125	312.5	
10 min	2.97	99.00	91	86	217.5	2.99	99.67	131	128	320	
15 min.	2.95	98.33	79	76	192.5	2.98	99.33	110	105	265	
30 min.	2.94	98.00	59	53	101	2.97	99.00	79	74	198	
1 h	2.95	98.33	38	35	87.5	2.95	98.33	45	42	105	
2 h	2.93	97.67	29	23	63	2.89	96.33	20	16	42.5	
24 h	2.92	97.33	24	19	50	2.86	95.33	12	9	25	

**Table 3:** Potentiometric selectivity coefficients of some interfering ions using CMCPEs (electrodes III and A) and MSPEs (electrodes VII and B).

**Interfering ions (B)**	**K**_LCHC__, B _ **(** **SSM)**			
	**MCPE**		**MSPE**	
	**III**	**A**	**VII**	**B**
Cr^3+^	6.1×10^-5^	4.7 ×10^-6^	3.2×10^-5^	3.5×10^-6^
Ba^2+^	4.4×10^-5^	2.6×10^-5^	2.1×10^-5^	1.1×10^-5^
${\mathrm{NH}}_{4}^{+}$	8.8×10^-6^	5.2×10^-6^	3.5×10^-6^	1.9×10^-6^
Co^2+^	9.4×10^-5^	7.4×10^-6^	6.5×10^-5^	5.7×10^-6^
Mg^2+^	5.7×10^-5^	8.2×10^-6^	4.8×10^-5^	7.6×10^-6^
Ca^2+^	6.6×10^-6^	5.9×10^-6^	4.3×10^-6^	4.0×10^-6^
Zn^2+^	7.5×10^-6^	5.1×10^-6^	6.8×10^-6^	9.3×10^-7^
Cu^2+^	8.1×10^-5^	8.0×10^-5^	6.3×10^-5^	5.3×10^-5^
Mn^2+^	4.6×10^-5^	2.4×10^-5^	7.2×10^-6^	3.8×10^-6^
**K**_LCHC__, B_ ** MPM**				
Glycine	4.9×10^-4^	3.2×10^-4^	1.7×10^-5^	0.9×10^-5^
Fructose	8.4×10^-3^	6.2×10^-3^	4.3×10^-3^	2.0×10^-3^
Glucose	7.4×10^-3^	5.0×10^-3^	2.2×10^-4^	1.0×10^-4^
Sucrose	3.2×10^-3^	6.2×10^-3^	2.4×10^-4^	5.9×10^-4^
Maltose	0.5×10^-4^	1.5×10^-4^	3.2×10^-4^	6.8×10^-4^
Lactose	7.1×10^-3^	6.5×10^-3^	4.7×10^-3^	2.7×10^-3^
Starch	8.6×10^-3^	7.0×10^-3^	5.3×10^-3^	1.4×10^-3^

**Table 4:** Potentiometric determination of LCHC in pharmaceutical formulations using MCPEs (electrodes III and A) and MSPEs (electrodes VII and B).

**Sample** **No.**	**[LCHC] mg/mL** ** Pharmaceutical Preparation**					**RSD(%)**				
	**British Pharmacopeia**	**III**	**A**	**VII**	**B**	**British Pharmacopeia**	**III**	**A**	**VII**	**B**
1	0.496	0.495	0.498	0.497	0.503	0.983	1.007	0.647	0.527	0.236
2	0.493	0.495	0.497	0.499	0.510	0.957	1.064	1.036	0.136	0.095
3	0.990	0.994	0.996	0.999	0.998	1.045	1.074	0.973	0.562	0.747

SD values for Pharmaceutical Preparation (British Pharmacopeia = 0.213-0.789), (electrode III = 0.154-0.634) (electrode A = 0.125-0.603), (electrode VII = 0.073- 0.562) and (electrode B = 0.015- 0.078).

**F-test** = (electrode III = 1.7 – 2.1), (electrode A = 0.5 – 1.2), (electrode VII = 0.3 – 1.0) and (electrode B = 0.09 – 0.8). (Tabulated F value at 95% confidence limit = 6.39 for n = 4).

**t-test** = (electrode III = 1.8 – 2.3), (electrode A = 0.7 – 2.1), (electrode VII = 0.6 – 1.8) and (electrode B = 0.12 – 1.2). (Tabulated t value at 95% confidence limit = 2.776 for n = 4).

Samples 1, 2 and 3 were lidocaine gel, farco-Caine oint and lidosine oint, respectively.

**Table 5:** Determination of LCHC in spiked urine and human serum using MCPEs (Sensor III) and MSPEs (Sensor VII).

**Sample**	**Statistical** **parameters**	**(Sensor III)**			**(Sensor VII)**		
		**Direct** **method**	**Calibration graphs**	**Standard addition** **method**	**Direct** **method**	**Calibration graphs**	**Standard addition** **method**
urine	Mean recovery (%)	99.12	98.77	97.98	99.60	99.00	98.80
	N	5	5	5	5	5	5
	Variance	0.85	0.72	0.80	0.78	0.57	0.63
	RSD (%)	0.38	0.53	0.81	0.42	0.51	0.67
serum	Mean recovery (%)	99.44	99.13	99.00	98.98	99.21	97.78
	N	5	5	5	5	5	5
	Variance	0.47	0.56	0.72	0.34	0.29	0.49
	RSD (%)	0.62	0.53	0.81	0.44	0.37	0.65

**Table 6:** Evaluation of intra- and inter-days precision and accuracy of MCPEs (electrodes III and A) and MSPEs (electrodes VII and B) in Pharmaceutical Preparation and water samples.

**Electrode type (plasticizer used)**	**Sample No.**	**[LCHC] ** **Taken,** **(mg/mL)**	**Intra day**				**Inter day**			
			**[LCHC]** **Found, (mg/mL)**	**Recovery** * **(%)**	**SD**	**RSD%**	**[LCHC]** **Found, (mg/mL)**	**Recovery** * **(%)**	**SD**	**RSD%**
III (TCP)	Pure LCHC	0.25	0.249	99.60	0.009	0.563	0.248	99.20	0.012	0.753
		0.50	0.498	99.60	0.007	0.215	0.495	99.00	0.003	0.895
	Sample 2	0.50	0.495	99.00	0.011	0.346	0.494	98.80	0.052	0.579
		1.00	0.995	99.50	0.013	0.532	0.996	99.60	0.041	0.421
	Sample 3	0.50	0.491	98.20	0.126	1.062	0.490	98.00	0.089	0.989
		1.00	0.994	99.40	0.012	0.361	0.992	99.20	0.037	1.035
A (*o*-NPOE)	Pure LCHC	0.25	0.248	99.20	0.013	0.458	0.247	98.80	0.153	0.892
		0.50	0.497	99.40	0.037	0.857	0.496	99.20	0.816	0.937
	Sample 2	0.25	0.243	97.20	0.062	1.521	0.240	96.00	0.062	2.024
		0.50	0.492	98.40	0.048	1.253	0.491	98.20	0.073	2.009
	Sample 3	0.50	0.493	98.60	0.028	1.012	0.490	98.00	0.098	1.014
		1.00	0.991	99.10	0.009	0.162	0.993	99.30	0.003	0.098
VII (TCP)	Pure LCHC	0.25	0.249	99.60	0.009	0.087	0.251	100.4	0.005	0.173
		0.50	0.501	100.2	0.003	0.058	0.500	100.0	0.002	0.132
	Sample 2	0.50	0.490	98.00	0.013	0.910	0.488	97.60	0.073	1.953
		1.00	0.989	98.90	0.062	1.006	0.985	98.50	0.036	1.457
	Sample 3	0.50	0.489	97.80	0.931	1.424	0.487	97.40	0.875	1.741
		1.00	0.988	98.80	0.427	1.842	0.986	98.60	0.655	1.952
B (*o*-NPOE)	Pure LCHC	0.25	0.249	99.60	0.010	0.173	0.248	99.20	0.012	0.967
		0.50	0.499	99.80	0.005	0.098	0.502	100.4	0.003	0.084
	Sample 2	0.50	0.479	95.80	0.063	1.261	0.486	97.20	0.032	1.077
		1.00	0.987	98.70	0.162	1.075	0.985	98.50	0.043	1.025
	Sample 3	0.50	0.491	98.20	0.091	0.936	0.489	97.80	0.668	1.067
		1.00	0.993	99.30	0.002	0.231	0.991	99.10	0.036	0.993

Sample 2 was farco-Caine oint.

Sample 3 was lidosine oint.

* Number of replicates = 5.

**Table 7:** Comparing some of the LCHC-MCPE (electrode III) and LCHC-MSPE (electrode VII) characteristics with some of the previously reported LCHC-ISEs.

**References**	**Slope** **(mV decade** ^-1^ **)**	**Response** **time (s)**	**pH**	**Life time (months)**	**Linear range (mol L** ^-1^ **)**	**DL (mol L** ^-1^ **)**
Proposed electrode (electrode III)	57.50	6	2.0-7.5	4	6.2×10^-7^ - 1×10^-2^	6.2×10^-7^
Proposed electrode (electrode VII)	58.90	4	‎2.0-8.0‎	6	1.0 × 10^-7^ – 1.0 × 10^-2^	1.0 × 10^-7^
37	60.10	< 10	5.0 - 9.5	3.5	1.0 × 10^-4^ - 1.0 × 10^-1^	6.3 × 10^−5^
38	57.10	< 10	2.0 – 8.0	6	1.0 × 10^-5^ – 1.0 × 10^-3^	2.0 × 10^-6^
39 (Electrode A)	58.20	-	2.0-7.5	-	1.0 × 10^-4^ - 1.0 × 10^-1^	2.5 × 10^-5^
39 (Electrode B)	57.30	-	2.0-7.5	-	3.2 × 10^-5^ - 1.0 × 10^-1^	1.0 × 10^-5^

*Effect of soaking time*

The surface of freshly prepared electrodes must be soaked to activate it through the formation of an infinitesimally thin gel layer at which ion exchange occurs. This preconditioning process required different times depending on diffusion and equilibration at the electrode-test solution interface. The effect of soaking time on the electrode performance was evaluated by soaking freshly prepared electrodes in [LCHC:NaTPB] suspended solution for 0 (without), 5, 10, 15, 60 and 120 min and 24 h to form a thin gel layer at which the ion exchange occurred. The optimum soaking time was found to be 0 and 10 min for MCPE and MSPE, respectively, where the highest total potential change and the potential break at the end point were obtained at 25 ˚C. They decreased with increasing soaking time (Table 2). Soaking for longer time than 0 and 10 min was not recommended for MCPE (electrode III) and MSPE (electrode VII), respectively, to avoid leaching of, although very little electroactive species into the bathing solutions but soaking was not recommended for MCPEs.

*Effect of pH*

The electrode response for LCHC solution was tested at different pH values (pH 1–11). The pH value was adjusted by adding very small volumes of HCl and/or NaOH solution (0.1–1 mol L^-1^ of each) to 3 mL of the LCHC solution, E (mV) versus pH values were then plotted and the potential of the electrode was plotted against the pH of solution. The results obtained indicated that the response of the electrodes was pH independent in the pH range 2.0 – 7.5 and 2.0 – 7.5 for MCPEs with tricresylphosphate (TCP) (electrode III) and *o*-nitrophenyloctylether (*o*-NPOE) (electrode A) as plasticizers, respectively. Also, the pH range 2.0-8.0 and 2.0-8.0 for MSPEs with tricresylphosphate (TCP) (electrode VII) and *o*-.nitrophenyloctylether (*o*-NPOE) (electrode B) as plasticizers, respectively. The decrease in mV readings at pH < 2 may be due to interference of hydronium ion. At higher pH values (pH > 8.0), free-base precipitated in the test solution and consequently, the concentration of unprotonated species gradually increased. As a result, lower e.m.f. readings were recorded as shown in Figure 4.

*Effect of Temperature of the Test Solution*

Calibration graphs (electrode potential (E_elec_) versus p[LCHC]) were constructed at different test solution temperatures (20–60 ˚C) using MCPE and MSPE. For the determination of the isothermal coefficient (dE^o^/dt) of the electrode, the standard electrode potentials (E^o^) against the normal hydrogen electrode at different temperatures were obtained from calibration graphs as the intercepts at p[LCHC] = 0 (after subtracting the values of the standard electrode potential of the silver-silver chloride double-junction reference electrode at these temperatures) and were plotted versus (t-25), where t was the temperature of the test solution in ˚C. A straight-line plot was obtained according to Antropov’s equation:

E^o ^= E^o^(25) + (dE˚/dt) (t − 25)

where E^o^(25) is the standard electrode potential at 25 ^o^C, the slope of the straight-line obtained represents the isothermal coefficient of the electrodes (0.00122, 0.00109, 0.00045 and 0.00028 mV/ºC) for electrodes (III), (A), (VII) and (B), respectively (Figure 5). The value of the obtained isothermal coefficient of the electrodes indicated that the electrodes had fairly high thermal stability within the investigated temperature range. The investigated electrodes were found to be usable up to 60 ˚C without noticeable deviation from the Nernstian behavior.

*Selectivity coefficient*

Selectivity is an important characteristic, which defines the nature of the device and the range to which it may be successfully employed. The selectivity of the ion selective electrodes under consideration were also, investigated with respect to some common cations using SSM. The data obtained (Table 3) showed the selectivity coefficients (K^pot^_A, B_) values for the tested cations. These values clearly indicated that, the proposed electrodes were fairly selective to lidocaine cation over different tested cations. Nevertheless, for all of the diverse ions used, the selectivity coefficients were lower than 1, that the studied common cations would not significantly disturb the determination of lidocaine. According to the SSM, the potentiometric selectivity coefficients were determined using 1 ×10^-3 ^mol L^-1^ test solution of different cations at pH = 5. The resulting selectivity coefficients are summarized in Table 3.

logK^pot^_A, B_ = ((E_B_-E_A_)/S) + (1-(Z_A_/Z_B_)) log a_A_

where, E_A_ is the potential measured in 1×10^-3^ mol L^-1^ LCHC (A), E_B_ the potential measured in 1 × 10^-3^ mol L^-1^ of the interfering compound (B), Z_A_ and Z_B_ are the charges of the LCHC (A) and interfering species (B), respectively, and S is slope of the electrode selectivity coefficients of the lidocaine selective electrodes calculated by the modified separate solution method at 25 ˚C. 

While the selectivity coefficients for many nitrogenous compounds such as starch, sugars and glycine were obtained by the matched method which was totally independent on the Nicolsky equation. The following equation was applied:

K^pot ^_LDHC, B_= ( a ´_ LCHC_ – a_ LCHC_ ) / a_B_

The influence of some inorganic cations, anions, sugars and glycine on the LCHC-electrodes was investigated (Table 3). The selectivity coefficients values of the electrodes III, A, VII and B reflected a very high selectivity of the investigated electrodes for the LCHC cation. The inorganic cations did not interfere owing to the differences in ionic size, and consequently their mobilities and permeability, as compared with those of LCHC^+ ^(Table 3). Also, the smaller the energy of hydration of the cation, the greater the response of the paste will be. In case of sugars and glycine, the high selectivity was mainly attributed to the difference in polarity and lipophilic character of their molecules relative to LCHC.

*Response Time*

The electrode response time was evaluated by measuring the average time required for the electrode to reach a steady potential reading when the concentration (19, 20, 36) of the LCHC was suddenly increased from 10^-6^ to 10^-3^ mol L^-1^ (Figure 6). The MCPEs and MSPEs showed very fast response times (9, 7, 6 and 4s for concentration 10^-3^ mol L^-1^, 11, 9, 7 and 6 s for lower concentration, for electrodes (III), (A), (VII) and (B), respectively ) which were shorter than the previously published drug sensors (19, 20) and the equilibrium potentials essentially remained constant for 15 min. These fast response times can be explained by the fact that these electrodes contain carbon particles surrounded by a very thin film of *o*-NPOE and acting as a conductor and the absence of the internal reference solution. This fast and stable potential reading was reflected on the time needed for complete titration process as it was only about 0–10 min. The other tested electrodes, except the screen printed and carbon paste electrodes, showed longer response time than the reported MSPEs and CPEs.

*Lifetime*

The average lifetime for most of the reported ion selective sensors in the range of 4 and 6 months for MCPE (electrode III) and MSPE (electrode VII), respectively, was an important factor. After this time the potential break at the end point of the sensors was decreased, and the detection limit was increased. The modified carbon paste electrodes reported here were tested for a period of 4 months, during which the electrodes were extensively studied. The modified SPE can be used for 6 months 

(Figure 7). It was obvious that at first, a slight gradual decrease in the potential break at the end point and, secondly, increases in the detection limit were observed. 

The reason for this limited life times of the modified electrodes can be attributed to one of the following factors namely the loss of plasticizer, carrier, or ionic site from the polymeric film due to leaching into the sample.

*Analytical applications*

Speciﬁcity was the ability of the method to measure the analyte response in the presence of all the potential interference. The response of the analyte with excipients, were compared with the response of pure LCHC. The modified screen printed and carbon paste electrodes were fabricated as previously reported by the research group (14). In order to assess the validity of the prepared electrodes, the potentiometric titration methods were applied for the determination of LCHC in pharmaceutical preparation and water samples using MSPEs (electrodes III and B) and MCPEs (electrodes VII and A) plasticized with TCP and *o*-NPOE. The application of proposed method for the potentiometric determination of LCHC in pharmaceutical preparation samples gave good results as shown in (Table 4). The results obtained using MCPE were compared with those obtained with MSPE. It showed that the electrodes prepared by MSPE and MCPE method had good efﬁciency as regard of sensitivity, index of retrieving and repetition. The calculated t and F values were smaller than the tabulated values indicating no significant difference between the proposed and official method.

As the conventional method for determination of LCHC (titration in non-aqueous solvents) was difﬁcult and time-consuming, as well as using an expensive solvents, this method (potentiometric determination) was easy, fast and inexpensive. One of the important applications of these drug-selective electrodes would be using for routine measurement of LCHC determination.

*Application to urine and human serum*

The determination of LCHC in spiked urine and human serum samples was carried using the standard additions method. The mean recoveries obtained were in range of 97.98 - 99.12 and 98.80 - 99.60% for electrodes III and VII, respectively , in urine samples. But in case of serum samples the mean recoveries were found to be 99.00-99.44 and 97.78-99.21 for electrodes III and VII, respectively (Table 5). 

The proposed methods can therefore be applied to the determination of LCHC in pure drug, in pharmaceutical preparations, and also in spiked human serum and urine samples without fear of interference caused by the excipients or degradation product expected to be present in tablets or in the constituents of body ﬂuids.In order to determine the precision of the proposed potentiometric method, two different concentrations of pure LCHC solution and different pharmaceutical preparation samples (Table 4) or five different titration runs of 2 mL of 10^-2^ mol L^-1^ LCHC with 10^-2^ mol L^-1^ NaTPB solution (Table 6) were performed, in order to evaluate the reproducibility of the results obtained. Tables 4, 6 gave statistical summary of each of the titration series using the modified SPE and CPE sensors. RSD and SD values were obtained within the same day to evaluate repeatability (intra-day precision) and over five days to evaluate intermediate precision (inter-day precision). 

The low values of the relative standard deviation (RSD) and standard deviation (SD) also indicated the high precision and the good accuracy of the proposed method.

*Comparative study*

For comparative purposes, Table 7 lists the linear range, detection limit, slope, pH range and response time of recently published LCHC-selective electrodes against the proposed electrode (37-39). From the results in these Tables, it can be concluded that, in many cases, the performances of the proposed electrodes show superior behavior if compared with the previously reported electrodes.

## Conclusion

The potentiometric procedure proposed here eliminates the prior separation steps that were usually necessary in the determination of lidocaine (LCHC) in pharmaceutical preparations and biological fluids (urine and serum) samples. Additionally, the proposed method had some important advantages: the electrodes proved to be successful, providing a rapid, simple and low cost potentiometric method for the determination of lidocaine in pure solutions, in pharmaceutical preparations, urine, human serum and spiked real water samples. It ensured a good accuracy for the lidocaine assay due to the possibility to control the ion activity continuously and also a fast assay of lidocaine oint.
